# Intralesional corticosteroid injection technique for scarring and nonscarring alopecia: A cross-sectional survey of hair experts in the United States

**DOI:** 10.1016/j.jdin.2025.04.007

**Published:** 2025-03-06

**Authors:** Noelle Desir, Iain Noel Encarnacion, Oluwatomilola Oyasiji, Kevin Puerta Durango, Charissa N. Obeng-Nyarko, Temitayo Ogunleye, Susan C. Taylor

**Affiliations:** a Department of Dermatology, Perelman School of Medicine at the University of Pennsylvania, Philadelphia, Pennsylvania; b Weill Cornell Medical College, New York, New York; c Eastern Virginia Medical School, Norfolk, Virginia; d Wayne State University School of Medicine, Detroit, Michigan; e Geisel School of Medicine at Dartmouth College, Hanover, New Hampshire; f Florida State University College of Medicine, Tallahassee, Florida

**Keywords:** alopecia, facial alopecia, general dermatology, hair, medical dermatology, nonscarring alopecia, scarring alopecia

*To the Editor:* Intralesional corticosteroids (ILCs), specifically triamcinolone, are a mainstay therapeutic for many alopecia types.^1^, ^2^, ^3^ Despite its cornerstone status, there are no standardized guidelines, leaving room for technique variability amongst dermatologists. To evaluate the potential need for a Delphi study, we surveyed academic dermatologists that specialize in hair on their ILC technique for alopecia. Our results of compiled details regarding ILC procedure technique from surveyed experts may provide additional guidance in the absence of a Delphi.

A 77 question REDCap survey inquired practicing dermatologists about demographic information and ILC technique. The survey was distributed via email to 28 preselected dermatologists, identified by authors’ personal knowledge of dermatologists with hair expertise. Descriptive statistics were performed in REDCap.

Three quarters of hair experts (21/28) completed the survey (response rate 75%). Respondents completed residency between 1985 and 2022 averaging 17.7 years (SD 12.2) of experience. For nonscarring alopecia, 18 of 21 (85.7%) use a concentration of 5 mg/mL, 20 of 21 (95.2%) inject between 1-1.5 mL per session, 20 of 21 (95.2%) use a 30-gage needle, 17 of 21 (81.0%) use a ½-inch needle, 20 of 21 (95.2%) inject in a grid pattern, 14 of 21 (66.7%) space injection points by 1 cm, 19 of 21 (90.5%) inject to the depth of the dermis, 11 of 21 (52.4%) typically perform 6 sessions with 16 of 21 (76.2%) denoting there is no maximum number of sessions, and the preferred treatment interval for 13 of 21 (61.9%) was every 6 weeks (Tables I and II). 15 of 21 (71.4%) experts have identical ILC technique between nonscarring and scarring alopecia. Likewise, facial alopecia ILC techniques were like those of nonscarring; key differences are that 16 of 20 (80.0%) use a concentration of 2.5 mg/mL, 13 of 20 (65.0%) inject between 1-1.5 mL per session and 18 of 20 (90%) typically perform between 3-4 sessions (Tables I and II).

**Table I:** Intralesional corticosteroid injection parameters employed by surveyed experts (Part 1)

Injection parameters	Nonscarring alopecia, *n* (%) (*N* = 21)∗	Scarring alopecia, *n* (%) (*N* = 5)∗^,^†	Facial alopecia, *n* (%) (*N* = 20)∗^,^‡
Concentration used (mg/mL)			
1	1 (4.8)	0 (0.0)	0 (0.0)
2.5	8 (38.1)	1 (20.0)	16 (80.0)
5	18 (85.7)	3 (60.0)	7 (35.0)
7.5	1 (4.8)	2 (40.0)	0 (0.0)
10 or more	5 (23.8)	2 (40.0)	0 (0.0)
Volume used per session (mL)			
<1	4 (19.0)	0 (0.0)	9 (45.0)
1-1.5	20 (95.2)	2 (40.0)	13 (65.0)
2-2.5	14 (66.7)	3 (60.0)	3 (15.0)
3-4	14 (66.7)	3 (60.0)	1 (5.0)
5-7	3 (14.3)	0 (0.0)	0 (0.0)
8 or more	1 (4.8)	0 (0.0)	0 (0.0)
Other: Varies by area	2 (9.5)	0 (0.0)	2 (9.5)
Needle gauge			
30	20 (95.2)	4 (80.0)	19 (95.0)
32	1 (4.8)	0 (0.0)	1 (5.0)
No response	0 (0.0)	0 (0.0)	2 (9.5)
Needle length (in)			
5/16	1 (4.8)	0 (0.0)	1 (5.0)
1/2	17 (81.0)	4 (80.0)	14 (70.0)
3/4	0 (0.0)	0 (0.0)	1 (5.0)
1	0 (0.0)	0 (0.0)	1 (5.0)
No preference	4 (19.0)	1 (20.0)	3 (15.0)
Injection pattern			
Grid pattern	20 (95.2)	3 (60.0)	12 (60.0)
Other: Targeted approach	1 (4.5)	2 (40.0)	8 (40.0)
Distance between needle insertions (cm)			
0.5	8 (38.1)	2 (40.0)	12 (60.0)
1	14 (66.7)	3 (60.0)	10 (50.0)
1.5	3 (14.3)	0 (0.0)	0 (0.0)
Other: Closer on eyebrows	1 (4.8)	0 (0.0)	0 (0.0)

∗ Respondents selected ≥1 answer choice.

† 15/21 (71.4%) had identical ILC technique for scarring and nonscarring alopecia. *N* = 5 represents the 5/21 (23.8%) that had technique differences between scarring and nonscarring alopecia.

‡ One expert abstained from questions regarding the use of ILC for facial alopecia because they do not use ILCs on the face.

**Table II:** Intralesional corticosteroid injection parameters employed by surveyed experts (Part 2)

Injection parameters	Nonscarring alopecia, *n* (%) (*N* = 21)∗	Scarring alopecia, *n* (%) (*N* = 5)∗^,^†	Facial alopecia, *n* (%) (*N* = 20)∗^,^‡
Injection depth			
Epidermis	2 (9.5)	0 (0.0)	1 (5.0)
Dermis	19 (90.5%)	5 (100.0)	18 (90.0)
Subcutaneous	5 (23.8)	1 (20.0)	1 (5.0)
Other: Depends on body site or alopecia sub-type	2 (9.5)	0 (0.0)	0 (0.0)
Preferred treatment intervals			
Every 4 wk	11 (52.4)	2 (40.0)	7 (35.0)
Every 6 wk	13 (61.9)	3 (60.0)	11 (55.0)
Every 8 wk	7 (33.3)	1 (20.0)	8 (40.0)
Every 10 wk	1 (4.8)	0 (0.0)	1 (5.0)
Every 12 wk	3 (14.3)	1 (20.0)	3 (15.0)
Typical number of intralesional corticosteroids sessions performed			
3 of fewer	8 (38.1)	1 (20.0)	9 (45.0)
4	6 (28.6)	0 (0.0)	9 (45.0)
5	5 (23.8)	1 (20.0)	5 (25.0)
6	11 (52.4)	2 (40.0)	4 (20.0)
7	2 (9.5)	1 (20.0)	1 (5.0)
8	1 (4.8)	1 (20.0)	1 (5.0)
12 or more	3 (14.3)	1 (20.0)	0 (0.0)
Maximum number of sessions			
3	1 (4.8)	2 (40.0)	2 (10.0)
5	0 (0.0)	0 (0.0)	1 (5.0)
6	3 (14.3)	1 (20.0)	8 (40.0)
8	1 (4.8)	0 (0.0)	0 (0.0)
9	1 (4.8)	0 (0.0)	0 (0.0)
No maximum	16 (76.2)	3 (60.0)	9 (45.0)
Clinical pearls for nonscarring alopecia	Inject smaller amounts closer together.		
	The amount used per session varies significantly [from 1-6 mLs] depending on the number of affected areas.		
	[There is] less pain when the injection angle is parallel to hair shaft because the follicle is not bisected.		
	Ensure that the reconstituted mixture is well blended within the syringe.		
Clinical pearls for scarring alopecia	Perform peripheral injections first and work towards the center of the lesion.		
	For central centrifugal cicatricial alopecia, once the patient is asymptomatic for 2 months, switch to maintenance (2-4 times a year and increase as needed).		
Clinical pearls for facial alopecia	Avoid areas with obvious vessels to avoid atrophy/bruising.		
	Depth of injection may be less than scalp because epidermis and dermis is thinner.		

∗ Respondents selected ≥1 answer choice.

† 15/21 (71.4%) had identical ILC technique for scarring and nonscarring alopecia. *N* = 5 represents the 5/21 (23.8%) that had technique differences between scarring and nonscarring alopecia.

‡ One expert abstained from questions regarding the use of ILC for facial alopecia because they do not use ILCs on the face.

For the treatment of nonscarring alopecia, experts highlight that patients may experience less pain when the injection angle is parallel to the direction of hair growth which prevents bisecting the follicle (Table II). For scarring alopecia, experts emphasize performing peripheral injections first then working toward the center of the lesion, and for facial alopecia, they recommend reducing injection depth and avoiding areas with obvious vessels to avoid atrophy or bruising (Table II). Study limitations include the hair expert identification process and response bias.

Our findings reveal general agreement amongst experts on the use of ILCs for alopecia; however, variation remains in concentration, injection spacing, and treatment duration, especially for facial and scarring alopecia. Interestingly, a randomized trial including 4 subjects found that injection of triamcinolone 2.5, 5 or 10 mg/mL for limited, patchy alopecia areata conferred the same benefit.^4^ Our surveyed experts most often reported concentrations of 5 mg/mL for nonscarring alopecia. This dose discrepancy should be further explored to optimize treatment results while limiting unnecessary exposure to corticosteroids, though there are minimal systemic side effects with ILCs especially in the small volumes recommended by experts in this study.^5^ While a Delphi study could help standardize intralesional corticosteroid approaches, our findings currently provide practical guidance for dermatologists on intralesional corticosteroid techniques for the treatment of various alopecia types.

## Conflicts of interest

Dr Taylor has served as a consultant, advisory board member, and/or speaker for AbbVie, Arcutis, Armis Scientific, Avita, Beiersdorf, Biorez, Bristol-Myers Squibb, Cara Therapeutics, Dior, Eli Lilly, EPI Health, Evolus, Galderma, GloGetter, Hugel America, Incyte, Johnson & Johnson, L’Oreal USA, MedScape, MJH LifeSciences, Pfizer, Piction Health, Sanofi, Scientis US, UCB, and Vichy Laboratories. She has received royalties from McGraw-Hill. She has served as an investigator for Allergan, Concert Pharmaceuticals/Sun Pharma, Croma-Pharma GmbH, Eli Lilly, and Pfizer. Dr Ogunleye has served as an advisory board member for Beiersdorf. Authors Desir, Encarnacion, Oyasiji, Puerta Durango, and Obeng-Nyarko have no conflicts of interest to declare.
